# Moral engine model: conceptualizing moral injury as a wound to the self caused by rupturing of defining and sustaining emotional attachments

**DOI:** 10.3389/fpsyt.2026.1735274

**Published:** 2026-10-22

**Authors:** William P. Nash

**Affiliations:** Weston, CT, United States

**Keywords:** attachment, cultural evolution, moral development, moral injury, object relations, self, self psychology, selfobject

## Abstract

A theory is elaborated illuminating the nature of moral injury based on evolutionary biology and psychoanalytic object-relations and self psychology, which explains how the betrayal of a moral trust can produce not only the negative moral emotions and losses of social and spiritual engagement characteristic of moral injury, but also the entire spectrum of adverse peri-and post-traumatic stress states. After stating the rationale for a few starting assumptions, three premises on which this theory is built are articulated, and their empirical support reviewed. The first premise is that morality is innate, evolving in vertebrates over a half billion years to enable the formation and evolution of cultures. The second premise is that pre-wired moral capacities comprise four concurrent moral modes serving a hierarchy of four moral imperatives: survive, be respected, love and belong, and seek the greater good. The third premise is that prosocial moral behavior continuously depends on the availability of secure and nourishing emotional attachments of three types (valued, valuing, and kindred), and that the rupture of an important emotional attachment may cause a sudden loss of the functional integrity of the self, manifested as dissociation and the fragmentation of experience and behavior. A theory of personhood called the Moral Engine Model, based on these three premises, is then described, articulating the relationship between the availability of defining and sustaining emotional attachments and one’s ability to become the best version of oneself and to contribute to the greater good through love, work, play, and creation. Two clinical vignettes are offered as frameworks for exploring the effects of betrayals of trust by oneself or someone else on the availability of diverse defining and sustaining, internal and external emotional attachments. Moral mapping is introduced as a graphical tool for visualizing changes in emotional attachments caused by moral injury traumas. Finally, a few thoughts about the possible application of the model to clinical treatment are offered.

## 1 Introduction

The past 15 years have witnessed a worldwide explosion of publications, conferences, and trainings focusing on moral injury, a new way of understanding psychological trauma first proposed by Jonathan Shay in 1994 (1), but brought to the broader attention of mental health scientists by Litz and colleagues in 2009 (2) and established as an important target for military occupational stress control in 2010 (3). The voices participating in the growing global conversation about moral injury—perhaps most broadly defined as enduring, intangible harm resulting from the betrayal of a moral trust—represent a broad cross-section of society, including philosophers, theologians, educators, ministers, jurists, humanists, artists, leaders in business and government, and medical and mental health scientists and practitioners. Research in moral injury has thus far primarily targeted military and veteran populations (4) and healthcare workers (5), the latter whose occupational risk for moral injury garnered public attention during the worst of the recent COVID-19 pandemic (6), although moral injury has now also been studied in police, firefighters, paramedics, teachers, refugees, and other trauma-exposed groups (e.g., (7–11)). Exposure to potentially morally injurious events has been found to be common among first-responders and other public service personnel—67% across all occupations in a large recent meta-analysis (12)—and such exposures have been correlated with clinically significant post-traumatic stress disorder (PTSD) and its most serious comorbidities: depression, substance abuse, and suicide (e.g., (12–15)). Another chronic stress state that has been correlated with exposures to morally injurious events is occupational burnout, a critical drain on the quality and availability of healthcare and other social services worldwide (16, 17).

As at-risk communities and providers of mental health services have come to appreciate the magnitude of moral injury as a public health problem, interventions to prevent or treat moral injury have also proliferated globally. Doing nothing is not an option. But in his recent review of the state of moral injury science, Litz (18) cautioned that we have yet to establish the required foundation for developing interventions for moral injury that can be proven safe and effective: a comprehensive, empirically testable theory that explains how betrayals of a moral trust, by oneself or someone else, can cause the specific post-traumatic harms observed in persons exposed to morally injurious events. A solid theory about the nature of any health problem—its pathophysiology—is a prerequisite because interventions are unlikely to ever be proven effective unless they are built on logic models connecting each element of the intervention to known or probable pathological processes (19). Any intervention not built on such a logic model is really a shot in the dark.

This paper describes a novel, integrative theory about the nature of moral injury, based principally on evolutionary biology and psychoanalytic self psychology, which identifies the parts of a person that are injured by the betrayal of a moral trust, and explains how such injuries can produce not only the negative moral emotions and losses of trust characteristic of moral injury, but also the entire spectrum of post-traumatic stress states, from burnout to complex post-traumatic stress disorder (CPTSD, (20)), the most serious post-traumatic health outcome.

## 2 Starting assumptions

### 2.1 Moral injury causes PTSD

I begin with the assumption that moral injury is frequently the cause of chronic post-traumatic stress in adults. This was Shay’s original hypothesis about combat-related PTSD, clearly stated in *Achilles in Vietnam*: “Moral injury is an essential part of any combat trauma that leads to lifelong psychological injury. Veterans can usually recover from horror, fear, or grief as long as ‘what’s right’ hasn’t been violated” (1, p. 20). Several epidemiological studies have supported Shay’s hypothesis, including the longitudinal Marine Resiliency Study [MRS, (21)], which found that the only category of stressor that significantly predicted new PTSD in battalions of ground-combat Marines 8 months post-deployment was moral injury events, unlike combat events, aftermath of battle experiences, perceptions of danger, or family conflict stressors (22, 23). No study of which I am aware has yet refuted Shay’s hypothesis.

### 2.2 The American psychiatric association’s conception of PTSD is not a valid comparator

The diagnostic criteria for PTSD that first appeared in the 1980 3^rd^ Edition of the APA’s Diagnostic and Statistical Manual [DSM-III, (24)] were drawn from published reports of two post-traumatic stress syndromes that emerged in the 1960s and 70s—Rape Trauma Syndrome [RTS, (25)] and Post-Vietnam Syndrome [PVS, (26)]—but with notable exceptions. Because the mental health professionals who drafted the original PTSD criteria believed that it could only be caused by experiences of intense physical fear, they ignored the symptoms of intense shame and anger that were prominent in both RTS and PVS. They also excluded from consideration as possible precipitants all potentially traumatic events not involving fear of death or severe physical injury. The five revisions of the APA’s diagnostic criteria for PTSD since DSM-III betray a retreat from the fear conditioning theory of trauma, but without acknowledging moral injury (or anything else) as an alternative causation, and without restoring shame and anger to their proper place as primary symptoms of enduring emotional trauma.

### 2.3 A moral injury is an information injury

Whatever the mechanism is by which experiences of moral betrayal cause enduring emotional harms, the medium through which these wounds are inflicted is information. A morally injurious experience is a pernicious moral message, which in its reception and processing is subject to all the physical constraints of any other information flow identified by Shannon (27), including limitations due to bandwidth and noise. This assumption directs attention in a theory of mechanism toward the parts of a person that receive and process social and spiritual information as likely sites of injury from moral messages. It also explains how traumatic experiences can be communicated from person to person and encoded into unconscious individual and communal beliefs, thus spreading individual moral injuries to families and communities [e.g., (28)].

### 2.4 Innate moral emotions enable cultural evolution

Charles Darwin was among the first to propose that emotions in humans and other animals represent a stream of involuntary appraisals of experience, based on social learning but driven by pre-wired perceive-and-respond programs, communicated to the experiencer through subjective physical feelings, judgments, and motivations, and to others through facial expressions, vocalizations, and other behaviors that are specific for each category of emotional experience, species, and individual, yet comprehensible across individuals and species (29). I argue that moral emotions represent a subset of pre-wired emotional experiences arising from unconscious appraisals of the rightness or wrongness of interactions with other moral agents or with oneself as a moral agent.

What can the survival value of the innate language of moral emotions possibly be? The more primitive instincts we share with fish are quicker because they bypass conscious awareness. If you touch a hot stove, you will have pulled your hand away before you are even aware of the pain, let alone have time to become angry with someone for leaving it on, or yourself for not checking. How have anger and shame, two principal moral emotions, aided the survival of Homo sapiens?

In 1976, the biologist Richard Dawkins upended evolutionary science by proposing that genes don’t care if any single individual survives or reproduces (the Darwinian *selfish organism* concept of survival of the fittest) as long as the gene, itself, survives and replicates (30). This was Dawkins’ *selfish gene* idea, which explains the evolutionary advantages of societies of all types, from ant colonies to the United Nations. Dawkins went on to propose that the societal effects of genes comprise their *extended phenotypes* (31), just as bipedal locomotion and opposable thumbs are part of the non-extended phenotype of humans as individuals. The capacity to feel and express emotions is clearly part of our extended phenotype and a major step in biological evolution toward the ultimate expression of the extended phenotype in any species: culture.

At its simplest, culture is merely the behavioral transfer of information between individuals through mimicry and teaching (32), but the advance of diverse human cultures has required more of their members than merely sharing information. The shared values, beliefs, norms, languages, technologies, and objects that comprise human cultures are growing and changing at an accelerating rate, no matter how bitterly anyone objects, continuously altering the human extended phenotype through the process of cultural evolution (30). Analogous to genetic evolution, cultural evolution adds to and modifies the information available to species members about the world and how to prosper in it, only obtained through a public competition of ideas rather than a private competition for survival or mating success. Cultural evolution accomplishes the same goal as genetic evolution, but millions of times faster, long-ago eclipsing natural selection through competition for survival or mating success as the principal shaper of human progress (30).

## 3 Innate morality: evolution, anatomy, development, and responses to stress

Before I can describe a theory about the nature of moral injury derived from evolutionary biology, I must first explain how innate moral emotions, motivations, and expectations might drive moral behaviors in humans and human societies, under normal circumstances, as the setting in which betrayals of moral trust and their consequences occur. Given popular conceptions of moral reasoning as an exclusively conscious process based on learned moral rules, it may be useful to examine how innate, emotion-driven morality might work differently.

Unfortunately, life sciences are only just beginning to work out how innate emotions work. Largely because prevailing theories of trauma have posited extreme fear of physical danger as the principal mechanism of harm, the only emotion that has been extensively studied across multiple species is fear (e.g., (33)). In this section, I present an admittedly speculative framework describing the anatomy and physiology of human social and moral functioning across the lifespan, integrating emerging findings from evolutionary neurophysiology, neuroendocrine, and functional neuroimaging studies. As will soon be apparent, tracing the evolution of social and moral emotions—the keys to understanding innate moral reasoning—suggests that moral information is not processed in a single control center in the human brain, but rather in four information processors operating simultaneously and semi-independently in different parts of the central nervous system (CNS), comprising four concurrent modes of moral reasoning in humans.

### 3.1 Four moral modes of the human central nervous system

**Figure 1** schematically depicts four levels of brain organization that evolved in vertebrates and persist in humans, as proposed by Paul MacLean at the National Institute of Health (34). MacLean referred to the human CNS as a *triune brain*, constructed in three distinct evolutionary steps—reptilian, paleomammalian, and neomammalian—but I have found it useful to separate the reptile brain from the earlier fish brain. For the sake of psychoeducation, I labeled the four human brain levels the Shark Brain, the Dragon Brain, the Wolf Brain, and the Angel Brain. **Table 1** characterizes the four moral modes hypothesized to be processed by these four levels of brain organization, each enabling a specific set of social capabilities driven by characteristic social and moral emotions serving an innate moral need.

**Figure 1:**
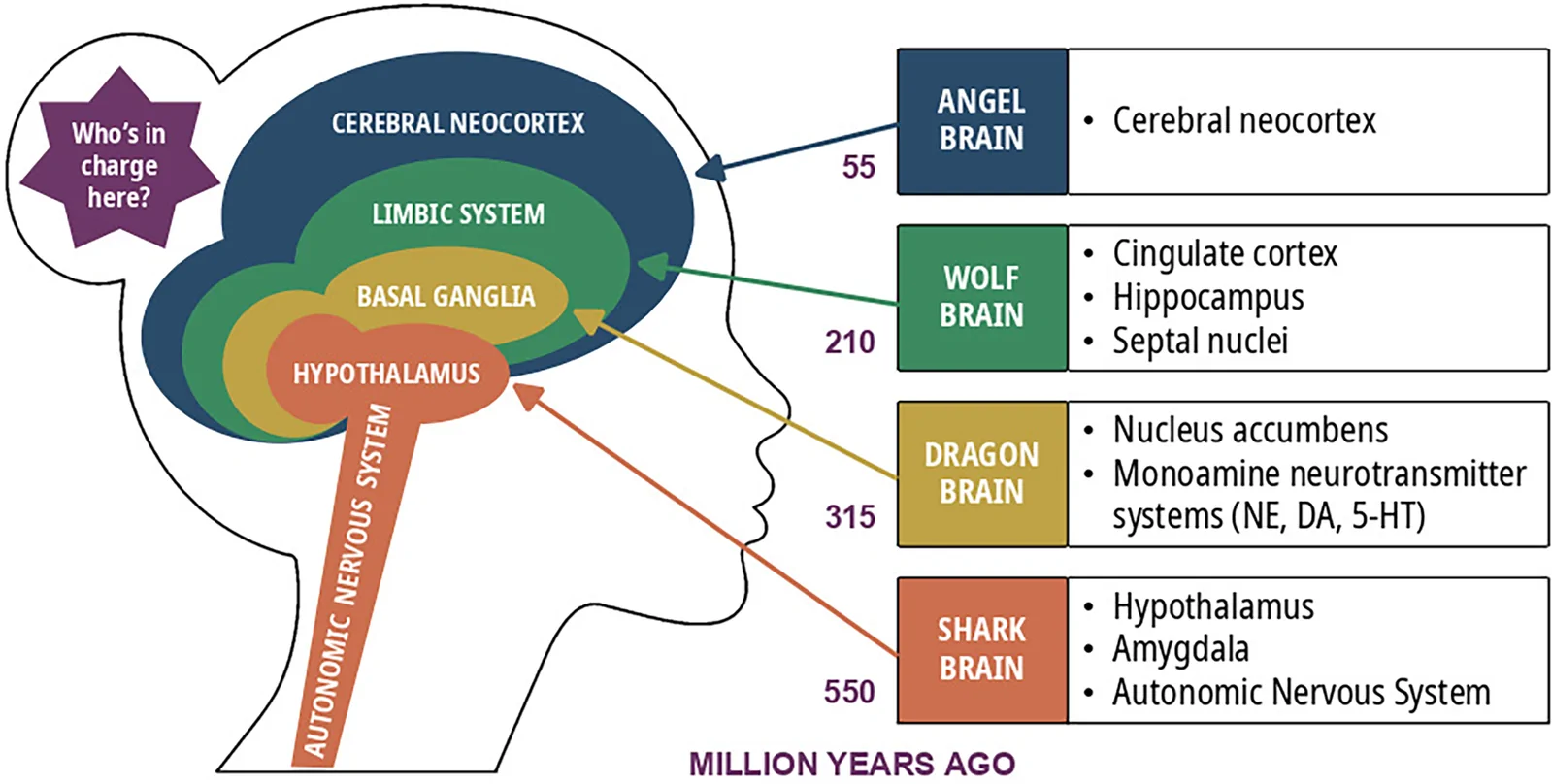
The four evolved levels of brain organization.

**Table 1:** Four evolved modes of innate morality.

	Major brain structures	Social capabilities	Social and moral emotions	Moral imperatives
Shark Brain	• Hypothalamus • Autonomic nervous system • Pituitary	• Schooling • Aggression • Predation • Mating	• Fear • Anger • Lust	• Survive • Reproduce
Dragon Brain	• Nucleus accumbens • Monoamine neurotransmitter systems (dopamine, norepinephrine, serotonin)	• Competition • Social hierarchies • Social displays • Leading and following	• Envy • Rage • Pride • Shame • Emotional pleasure • Emotional pain	• Acquire social power • Defend social rank • Hoard resources
Wolf Brain	• Cingulate cortex • Hippocampus • Parahippocampal gyrus • Septal nuclei	• Attachment • Cooperation • Teaching • Play	• Love • Hate • Joy • Sorrow	• Love • Be loved • Belong
Angel Brain	• Cerebral neocortex	• Self-awareness • Theory of mind • Cognitive empathy • Sense of humor	• Gratitude • Awe, wonder • Laughter • Tears	• Seek truth • Be just • Become your best self

#### 3.1.1 Shark brain morality: survive at all costs

The structure in the human brain that corresponds to the major control center in the fish brain is the hypothalamus, along with its sensory inputs from the thalamus, appraisal inputs from a primitive amygdala, and two sets of controlling outputs to the entire body: neural controls transmitted through the autonomic nervous system and endocrine controls through the adjacent pituitary. The hypothalamus is not principally an organ of social engagement; its main job in all vertebrates is homeostasis and allostasis: adjusting internal states to meet environmental demands. Its effectiveness is a major reason for the evolutionary success of vertebrates.

Except when feeding or breeding, most fish have little interest in one another. Shoaling and schooling in fish, like flocking in birds, involve little communication between individuals and no control of one individual by another. One fundamental social capacity that has been consistently mapped to the hypothalamus in all species is aggression (35). Aggression, or the use of force to gain an end, serves diverse purposes in various species, but every form of aggressive behavior in every vertebrate is mediated by the hypothalamus, driving both the sympathetic and parasympathetic nervous systems and the hypothalamic-pituitary-adrenal (HPA) axis of endocrine control. Since the instinctive fight-or-flight response to danger is mediated entirely by the hypothalamus and sympathetic nervous system, it is likely a common experience in fish, though rare in healthy adult humans because of more recently evolved inhibitory controls.

#### 3.1.2 Dragon brain morality: acquire and defend social rank

Two advances appeared in the brains of ancient reptiles about 315 million years ago (mya) that enabled social hierarchies, the earliest form of social organization in vertebrates. Social hierarchies depend on several distinct but interrelated capacities, including the abilities to recognize rivals, compete for social rank through ritual displays and combat, agree on who has won each competition, and keep track of which individuals in the community are superior or inferior in rank, and therefore entitled to better or worse territories and chances to mate. The first of two advances in the early reptilian brain that made social hierarchies possible was a repurposing of three ancient control systems in the brainstem to coordinate experience and behavior in complex and rapidly changing social situations, each powered by an extensive network utilizing one of three monoamine neurotransmitters: norepinephrine (excitement), dopamine (focus), or serotonin (stress-buffering). The second advance in the early reptilian brain was the appearance of a pair of new masses of neurons in the forebrain, one on each side of the midline, densely innervated with dopamine neurons originating in the brainstem: the striatal complex or basal ganglia.

MacLean (34) demonstrated the connection between the striatal complex and social hierarchies in the green anole lizard by surgically ablating the striatal complex on only one side of the brain of each experimental animal, then observing differences in their social behaviors depending on which eye they saw potential rivals through. He repeated this experiment with squirrel monkeys, demonstrating that social hierarchies in primates also depend on the basal ganglia, which in humans comprise a dorsally situated caudate and putamen (important for coordination of complex motor behaviors) and a more ventral nucleus accumbens (important for motivation, pleasure, mood, and pain control).

Human competition for social power and rank, likely mediated by the basal ganglia, is driven by some of the most destructive of all human emotions—pride, shame, envy, scorn, rage, and the talionic impulse: the craving for revenge as the only way to right a wrong. The Dragon Brain’s authority comes from the nucleus accumbens, the part of the basal ganglia that motivates all behavior using its abilities to steer attention and generate emotional pleasure and pain as carrot and stick. Any time a person’s social standing, dignity, prestige, or wealth is threatened by a social interaction, their Dragon Brain may respond with intense anger or shame and motivations to either defend their social standing and possessions or to submit to a reduction in rank. In any setting, perhaps the only human emotional disposition that trumps the Dragon Brain’s defense of social standing is the Shark Brain’s protection of life.

#### 3.1.3 Wolf brain morality: love, be loved, belong

Reptiles were the first vertebrates capable of social competition through dominance hierarchies, but they lacked two other capacities necessary to form a culture: the motivation to cooperate with members of their own species, and an innate language to facilitate cooperation. Both of these capabilities first appeared about 225 mya in small, burrowing mammals, made possible by a set of newly evolved grey-matter structures, collectively called the limbic system (34), surrounding the margin (*limbus* in Latin) of the forward end of the brainstem. **Figure 2** schematically depicts the locations and emerging functions of major components of the human limbic system.

**Figure 2:**
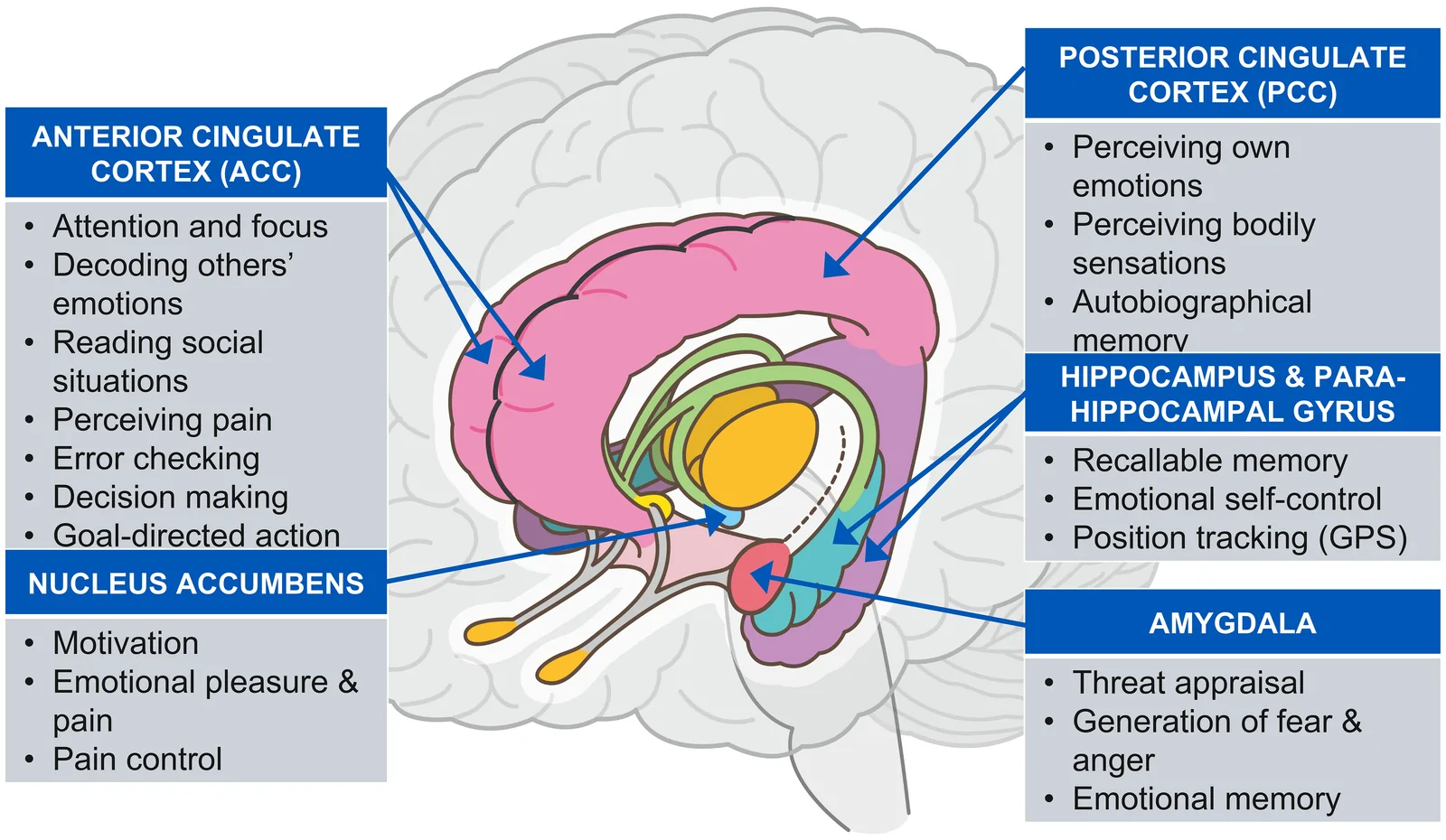
Major components of the human limbic system and their apparent functions.

Central to limbic system functioning are the left and right cingulate cortices: two parallel strips of grey matter, shaped in humans almost like two antelope horns, one on each side of the midline, curving backward over the white-matter band that connects the two cerebral hemispheres. Each cingulate cortex is divided into two interconnected gyri—two information processors on each side—processing two linked streams of social information in real time and in memory following experiences. At the forward end, the anterior cingulate cortex (ACC) attends to and decodes emotional information received from outside persons and objects (comprising one’s experience of the world), while at the same time but further aft, the posterior cingulate cortex (PCC) attends to one’s own emotions, motivations, and physical sensations during each interaction (comprising one’s experience of oneself).

The ACC and PCC appear to comprise a multitrack social memory system, continuously recording important experiences so they can be consciously recalled in the future, but also so they can be incorporated into personality as social procedural memories (see sec. 4.2). But the real mammalian superpower is what all this limbic machinery makes possible: the capacity for emotional attachment and love. Love provides the motivation while shared attachment provides the security necessary for long-term cooperation, parental nurturance, and the relatively prolonged periods of education and training necessary for many mammal species to reach their full adult potential. It also enables the level of long-term cooperation necessary for cultural evolution.

Moral emotions likely processed in the Wolf Brain include affection, devotion, warmth, compassion, and emotional empathy, but also feelings of guilt and hatred, love’s evil twin. The moral imperative of the Wolf Brain seems to be to nurture, protect, and commune with who and what one loves—to love, be loved, and belong (see sec. 4.2.2).

#### 3.1.4 Angel brain morality: seek the greatest good

The crowning achievement of the vertebrate brain, the neocortex, first appeared in early primates about 55 mya, enabling the development of the extensive capacities for conscious thought, symbolic communication, and self control required for cultural evolution. The most prominent social capacity enabled by the neocortex in humans is verbal language, whose grammar and syntax are pre-wired in language centers in the frontal and temporal lobes. Other social capacities that only occur in animals with large neocortices include self-awareness, cognitive empathy, a theory of mind (being able to imagine what someone else is thinking), an appreciation of music, and perhaps exclusively in humans, stand-up comedy. Two neocortical communication abilities that appear to fully exist only in humans are laughter and crying, two powerfully contagious emotional messages. Moral emotions processed by the Angel Brain likely include all those not originating in lower control centers, comprising some of the most transcendent states humans can experience: gratitude, awe, wonder, inspiration, and elevation. The moral imperatives of the Angel Brain likely depend less on hard-wired responses and more on learning of cultural standards and personal choices than the other three moral modes, but throughout human history, our highest moral aspirations have been to become the best versions of ourselves, to contribute to the greater good, to seek truth—a critical moral value for an organism constructed of information—and to embody virtue for its own sake (see sec. 4.1).

### 3.2 Human moral heterogeneity due to differences in development

Each of the four evolved moral modes likely traces its own developmental arc over each individual’s lifetime. Since the Shark Brain receives unfiltered sensory input from the entire body directly from the thalamus, it likely requires little learning or development to recognize and respond to threats to life. Rather, the greatest developmental challenge facing the Shark Brain, at least for people living in modern civilized societies, is to withhold reactions to minor dangers, to contain impulses to fight or flee every time threatened. Similarly, emotions, urges, and expectations arising in the Dragon Brain likely require little learning to be triggered in any situation in which one’s social worth and self-esteem are threatened; young children are capable of feeling deeply proud of their successes and ashamed of their perceived failures. The Dragon Brain’s greatest developmental challenge may be to learn to ride in the back seat—to resist the urges to dominate, steal, and exact revenge—to learn that the Dragon Brain’s self-esteem needs (and a whole lot more) can be better gained by cooperating with others in service of the greater good, as a member of a team bound by trust and guided by shared values.

In contrast, whereas we all likely begin life fully capable of emotional attachment, the ultimate social capacity that characterizes the Wolf Brain—agape love—can only be learned by experiencing it from others. Although little research yet informs this discussion, it seems likely that a prolonged (if not lifelong) period of development is required to fully develop the capacities of the Wolf Brain for joy in service to others. The Wolf Brain cannot meet its moral imperatives without the assistance of the prefrontal neocortex, the part of the Angel Brain that regulates emotions, thoughts, and behavior by focusing on what is important at the moment, while inhibiting all potentially distracting internal or external messages. The development of the neocortex, itself, is protracted because of a prolonged period of remodeling, pruning, and myelination extending from puberty into early adulthood.

### 3.3 Effects on moral functioning of extreme stress

If we had learned anything from the shell-shock epidemic of WWI, it should have been that normal, healthy people can be broken by extreme stress. Perhaps because it conflicts with idealized views of humans as unbound by biology, global cultures have been reluctant to embrace this truth. The first acknowledgment by the American Psychiatric Association that healthy people can become symptomatic and impaired solely because of excessive stress came in 1980 in DSM-III (24) with the introduction of adjustment disorder diagnoses, including a subtype called *adjustment disorder with disturbance of conduct*, characterized by stress-induced behaviors that violate the rights of others or societal norms. World militaries have long been aware that combatants under extreme stress can behave in ways that violate their own moral codes, including engaging in torture, rape, and premeditated murder, situations that have long been called *misconduct stress reactions* (36). In this section, I will briefly review research and theory exploring the effects of both unrelenting chronic stress and acute traumatic stress on brain function and moral reasoning.

#### 3.3.1 Unrelenting chronic stress and allostatic load

Emerging research has shown that chronic stress can damage top-down control systems in the brain through a common and important biological mechanism known as *allostatic load*. Allostasis is the normal process by which living systems adjust their rates of metabolism and other activities to meet current threats or demands. Common examples include an increase in heart rate during sustained exertion and a fever during an infection; both are situations in which a bodily system is stressed by an unrelenting challenge such that the original state of homeostasis is no longer possible. Allostasis always comes at a cost. The chemical messengers of stress—corticotropin releasing hormone, cortisol, epinephrine, and norepinephrine—are all toxic to living cells, and accelerated metabolism during allostasis causes cells to wear out faster and die sooner, triggering inflammation. The neuroendocrinologist Bruce McEwen coined the term allostatic load to refer to the damage caused to living systems by chronic stress (37).

McEwen showed how the allostatic load caused by chronic psychosocial stress in humans, such as that due to chronic illness or even just low socioeconomic status, could accelerate aging and the degeneration of nearly all bodily systems, leading to cardiovascular disease, metabolic disease like obesity or diabetes, immune dysfunction, cognitive decline, and premature death (38). More directly relevant to moral functioning, the allostatic load of chronic stress causes resources to be progressively shifted from neocortex to older, subcortical structures, causing top-down authority to progressively weaken at the same time bottom-up impulses like aggression become stronger. For example, brain-derived neurotrophic factor (BDNF), a stimulant for new neurons to grow from progenitor cells—to replace deceased neurons or to encode new long-term memories—is released more in subcortical structures like the amygdala and less in the hippocampus and neocortex during chronic stress, strengthening primitive emotions like fear and anger while progressively weakening cortical controls (39). Allostatic load may be one reason for Shark and Dragon modes of moral reasoning to predominate in chronically stressed individuals and impoverished communities, in whom more prosocial moral modes may be beyond reach.

#### 3.3.2 Acute traumatic stress and dissociation

The mechanism by which a single overwhelming stressor event can abruptly degrade brain functioning and moral reasoning is less well understood and far more controversial, even though we have known about peritraumatic dissociation—the tendency of people experiencing extreme levels of stress to lose control over parts of their bodies or minds—since it was first described in the mid-nineteenth century by the French neurologist, Moreau de Tours (40). Despite decades of subsequent research and its clear connection to common stress-related mental disorders from borderline personality disorder to PTSD, controversies endure regarding both the nature of dissociation and what symptoms may herald it. Here I will briefly describe three still-held theories about the nature of dissociation dating to the 1800s and explain why only one of them is consistent with evolutionary neuroscience and the model of innate morality presented here.

The most well-known theory about dissociation might be that described by Breuer and Freud in their 1895 *Studies in Hysteria*, where they hypothesized that stress-related losses of function were due to the ability of the unconscious mind to defend consciousness against potentially destabilizing emotions and thoughts by converting them into physical symptoms as a way for them to be expressed outside of conscious awareness (41). To Freud, dissociation was a choice, even if made unconsciously—a natural, normal, and reversible process that left the individual none the worse for wear, just like, in his view, other common defense mechanisms like repression and rationalization. In contrast, in his 1889 *Psychological Automatism*, Pierre Janet asserted that dissociation is always a pathological process, reflecting a breakdown in information processing in the brain under stress, but only appearing in people who suffered from a preexisting constitutional deficit in their neural integrative capacity (42). Thus, neither Freud nor Janet seemed to believe that the parts of the CNS that support psychological function in normal people could be physically harmed by overuse or over-stimulation, like every other organ in the body.

An alternative, earlier theory about the nature of dissociation was held by the pioneering British neurologist, John Hughlings Jackson, who—influenced more by Spencer than Descartes—believed not only that the mind and self were entirely products of the brain, but that under overwhelming stress, mental functions dissolved in each individual in an order directly opposite to that in which they had developed over each person’s lifespan and evolved across species (43). Jackson attributed the observed order of dissolution of brain function under stress to the relative weakness of top-down controls, developed during each person’s lifetime, compared to the more genetically hard-wired connections within each hierarchical brain level. Unlike Freud or Janet, Jackson believed that psychological function depended entirely on a healthy CNS operating within acceptable stress limits, which when breached, caused information processing in the brain to abruptly degrade in anyone.

Because no dissociation theory has yet been ruled out by compelling evidence, all three described here continue to influence research, clinical practice, and public policy. For example, whereas the American Psychiatric Association’s current DSM-5-TR contains an entire chapter of dissociative mental disorders which, following Jackson or Janet, are “characterized by a disruption of and/or discontinuity in the normal integration of consciousness, memory, identity, emotion, perception, body representation, motor control, and behavior” [(44), p. 291], the current American Psychological Association dictionary of psychology defines dissociation as “a defense mechanism in which conflicting impulses are kept apart or threatening ideas and feelings are separated from the rest of the psyche” (45), following Freud. Like Jackson, the MEM views dissociation as an abrupt loss of functional connectivity in the CNS causing an involuntary loss of function, which can happen to anyone given sufficient stress, illness, or other triggering factor. If this weren’t true, the human brain would qualify as the only computer on the planet impervious to crashing.

Peritraumatic dissociation has been studied as a risk factor for PTSD since 1994, beginning with the Peritraumatic Dissociative Experiences Questionnaire (PDEQ, 46)), which assessed cognitive forms of dissociation such as derealization (feeling in a fog or unreal state) or depersonalization (feeling disconnected from oneself). A 2003 meta-analysis of studies of risk factors for PTSD in adults found that peritraumatic dissociation, as measured by the PDEQ, was the strongest correlate of chronic PTSD after exposure to traumatic events, predicting PTSD better than any of the preexisting risk factors studied, such as age, gender, or trauma history (47). Cognitive dissociation is the only form included in the diagnostic criteria for the dissociative subtype of PTSD in DSM-5-TR (44), even though recent research has shown an equally elevated risk for chronic PTSD in persons who experience emotional forms of peritraumatic dissociation, such as panic or sobbing (48), somatic forms of dissociation like the paralysis or tremors that characterized WWI shell shock cases (49), or moral forms of dissociation, such as a loss of the ability to feel remorse or a determination to exact revenge (50).

## 4 Moral engine model of personhood

Before I can describe a mechanism by which the betrayal of a moral trust can inflict lasting harm to a person, I must first define *person*. Scholars have explored what it means to be a person from countless perspectives since the beginning of human history, none of them clearly superior to all others. For the purpose of understanding how the betrayal of an innate moral trust can cause lasting physical harm, however, the most promising perspective may be that of the genes that encode human moral and culture-forming capacities. If we could somehow ask our DNA why these capabilities evolved in us, what might it say? Can we imagine a higher-order, genetically pre-programmed culture-forming function that might define humanness more fully? A few clues come from studies of how optimally functioning humans spend their lives.

### 4.1 Defining the outputs of human moral engines

One of the few mental health scientists to study healthy humans was Abraham Maslow, whose well-known Hierarchy of Needs theory resulted from researching the drives and motivations of successful adults in diverse cultures (51). Maslow found that people who are relatively healthy and whole are never satisfied and seldom at rest, but rather continuously strive to achieve goals. As soon as one objective is achieved, the next one is quickly set and tackled. Maslow’s well-known conclusion was that the needs toward which well-functioning humans strive lie along a hierarchy, with basic physiological needs supporting survival at the base, and the ultimate goal of high-functioning humans, self-actualization, perched at the top. What is the goal of self-actualization—of becoming the best and most complete and authentic versions of ourselves? I argue that our highest need is to develop and grow in order to best contribute to the greater good, on behalf of the people and things we care about. This is what matters, what fills our lives with joy. Many theorists have identified love, work, and play as important for experiencing happiness and positive mental health (52). I add a fourth method for contributing to the greater good: *creation*, the engine of cultural evolution.

Contributions to the greater good might be considered the defining cultural outputs of human beings, but we haven’t yet identified the inputs required to fuel human moral engines. It is likely that input requirements for human moral functioning exist because many aspects of the enterprise—from the journey to become the best version of oneself to acquiring the skills to meaningfully contribute to the greater good—are incredibly difficult. Our definition should explain why so many people in diverse cultures seem to hate more than they love, derive little satisfaction from work or play, and seek to destroy as much as they create. And from the perspective of the survival of our species, why didn’t we become extinct long ago, given the destructive power of a single rageful person? Might our extended phenotype include some sort of fail-safe against the power of evil to extinguish good? I argue that the answer to both these questions—why the objectives of personhood can be so elusive, and why cultural movements grounded in hate rather than love tend to be self-limiting—is the premise that the moral engine that defines every human person is fueled by one particularly scarce and fragile resource: loving and secure emotional attachments.

### 4.2 Emotional attachments fuel moral engines

#### 4.2.1 Attachment needs in childhood

The scientific study of emotional attachment in children was pioneered in Great Britain in the early twentieth century by Melanie Klein and Anna Freud, two psychoanalytically trained women who, unlike Anna’s father, saw the importance of the mother-infant bond for emotional development. They observed the interactions of mothers and infants, and using play as a medium to communicate, studied and treated children with emotional problems as young as two and a half, laying the foundations for child psychiatry and psychology. Their work was given an unintentional (and unfortunately tragic) boost on September 1^st^, 1939, two days before Britain and France declared war on Germany, when the British government launched Operation Pied Piper, a massive program to evacuate children and other vulnerable civilians to rural areas or overseas, away from London and other urban areas within range of the German blitz (53). Within months, 800,000 children were evacuated to receiving centers, nurseries, and foster homes, many of them under 5 years of age and unaccompanied by any family member. It’s hard to imagine being surprised that so many evacuated children were traumatized by their experiences, but at that point, mental health science had no conception of emotional trauma other than that possibly due to extreme fear, and no understanding that a person could be harmed by separation from a loved one. By treating many of those evacuated children and comparing their outcomes to those of children who were not evacuated, Anna Freud and her team of researchers showed that fear of being killed by bombs did less long-term harm to children than separation from their families and homes (54). For her part, Melanie Klein pioneered the psychoanalytic theory of object relations, still the most robust explanation for why secure emotional attachments are critically important during childhood (55).

In psychoanalysis, an *object* is any person or thing to which a human being can emotionally attach—an infinite set, given human propensities for falling in love. Object relations theory explains how important love relationships become internalized and organized to form a person’s internal object world: an internal community of representations of important others and oneself in relation to them, in current life, fantasy, and from every point in the past. The internal world not only provides a storehouse of recallable information about people and things, it also becomes organized into procedural memories that form the templates for all future relationships as the structure of personality. As the first to identify this fundamental role of early life relationships, Klein attracted many psychoanalytically trained followers, especially in Great Britain, each making major contributions to object-relations theory. They included pediatricians like Donald Winnicott (56), ministers like Harry Guntrip (57), and psychiatrists like Ronald Fairbairn (58) and John Bowlby, the latter whose trilogy remains the bible for understanding attachment, separation, and loss during childhood (59–61).

#### 4.2.2 Attachment needs in adulthood

Adult requirements for emotional attachment were most extensively studied by Heinz Kohut, the ground-breaking psychiatrist who developed psychoanalytic self psychology through his long-term treatments of adults with narcissistic personality disorder (62–64), a condition previously considered untreatable by psychoanalysts. Even though gained through his work with this narrow population, Kohut made several discoveries about the self he believed had profound implications for mental health and wellbeing for anyone at any age.

Kohut’s first insight was that all love relationships involve a blurring of the boundaries between self and other, such that certain experiences, identifications, and responsibilities belong to neither party, individually, but rather always to both, existing only in the shared space created by intimacy. He gave these conjoined relationships the name *selfobject* to highlight the blurring of the boundary between self and other and the crucial role these relationships play in defining and sustaining selfhood. His second insight was that the human relationship repertoire comprises three distinct types of selfobject attachment, which he termed *idealized*, *mirroring*, and *twinship* (64) based on how he experienced them in his treatments of narcissistic personalities. In their more common and less extreme forms, these three types of emotional attachment may be termed *valuing* (cherishing and wishing to honor and emulate someone or something else), *valued* (being cherished and honored by someone else), and *kindred* (sharing a social identity with someone else as a peer, sibling, or teammate). Kohut’s third insight, only articulated at the end of his life, was that all humans depend on selfobject attachments every day of their lives, from birth to death, and that health, wholeness, and social competencies depend on continuous access to defining and sustaining selfobject attachments ( (64), p. 47).

Evidence of the lifelong dependence we have on each other includes the universal craving for human contact known as object hunger, the destructive impacts on health and wellbeing of solitary confinement, and the fact that people who live highly solitary, isolated lives seem to experience little joy. More to the point of understanding moral injury traumas, yet more evidence of the normal human dependency on secure, loving attachments is what may be Kohut’s greatest insight: that the perceived sudden rupture of an important selfobject attachment typically results in an abrupt loss of the functional integrity of the self and the fragmentation of experience and behavior (i.e., dissociation) as immediate consequences (62–64). Adults with personality disorders may be particularly susceptible to falling apart under stress, but Kohut’s key finding was that the rupture of an important selfobject attachment can cause immediate and potentially lasting fragmentation of the self in anyone at any age—not just people with personality disorders. This may be the “smoking gun” linking the abrupt loss of emotional attachments directly to peritraumatic dissociation.

### 4.3 The Moral Engine Model (MEM)

My definition of *person* and theory of personhood, the MEM, is schematically depicted in **Figure 3**. The main objectives of personhood can be read from left to right across the top—to become the best version of oneself in order to optimally contribute to the greater good through love, work, play, and creation, on behalf of people and things that one cares about. The reason being a person is so challenging and social successes can be so elusive can be read from right to left across the bottom—the human moral engine is exclusively fueled by secure and loving valued, valuing, and kindred sustaining attachments. A few key features of the MEM bear brief mentioning before applying it to moral injury.

**Figure 3:**
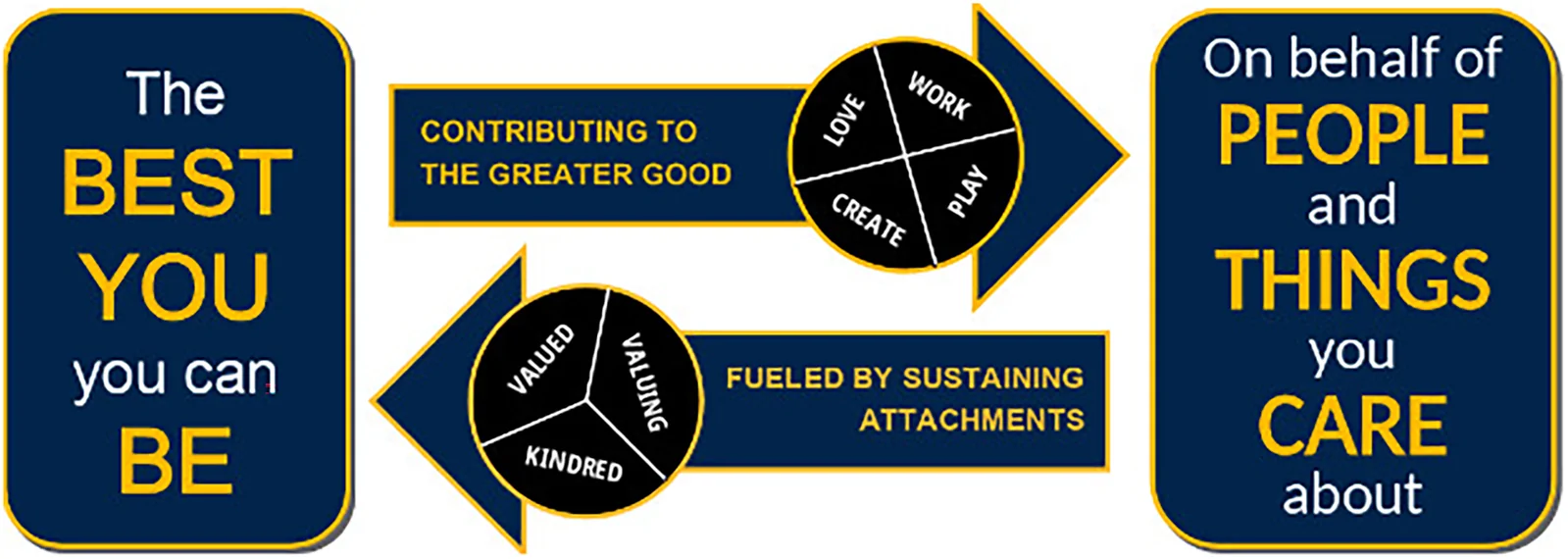
The moral engine model of personhood.

#### 4.3.1 The Moral Engine Model is experience-near

Kohut drew a critical distinction in his work on the nature of humanness between theories that he called *experience-near* rather than *experience-distant* ( (63), p. 303). He defined the former as theories built directly on (and successfully explaining) empathic observations of humans being human, made understandable by vicarious introspections accompanying those observations. In contrast, he defined experience-distant theories as those based on abstractions about humans rather than direct empathic observations. *Self* is a good example of an experience-near concept (unlike Freud’s *ego*, for example) because it requires no complex explanation to understand; when once asked what he meant by *self*, Kohut is said to have replied: “It means what *you* mean every time you say ‘I’ or ‘me’.”

#### 4.3.2 Emotional attachments are of countless diverse types, both external and internal

Applying the MEM to real-life experiences, especially those involving moral injury, requires an appreciation of the great numbers and types of people and things that can serve as defining and sustaining emotional attachments. It is impossible to name—or even imagine—them all, but **Table 2** proposes a two-by-two matrix for categorizing attachments, and offers examples of external and internal people and things that can serve as sustaining and defining emotional attachments.

**Table 2:** Categories and examples of people and things to which humans can establish defining and sustaining emotional attachments.

	PERSONS	NON-PERSONS
EXTERNAL	• Parents, siblings • Members of extended family • Romantic partners • Teachers, mentors • Friends • Coworkers, teammates • Leaders, followers • Cultural idols and icons • Public heroes • Exemplars • Role models	• Spiritual entities • Pets, other life forms • Cultures, ways of life • Physical places, locations • Possessions, wealth • Institutions • Professions • Arts, crafts, hobbies, sports • Ceremonies, rituals • Drugs, alcohol, food • Social power, money
INTERNAL	• Internalized current loved ones • Internalized past loved ones • Heroes from history or literature • Idealized images of oneself • Fantasies of ideal love	• Values, ideals • One’s own body • Personal strengths, gifts • Social skills & competences • Accomplishments

##### 4.3.2.1 External persons: our most important attachments

The most prominent category of emotional attachments is to other humans with whom we share our lives, such as parents, siblings, teachers, romantic partners, friends, and others listed in the upper-left quadrant of **Table 2**. External living persons are important emotional attachments because of their crucial role throughout life for the development of a rich and nourishing internal object world, necessary for both becoming the best version of oneself and for learning how to love, work, play, and create. Living humans likely comprise most valuing attachments—because except for a few particularly moral institutions, most nonliving objects do not return our love—as well as all kindred attachments, which by definition, are other people with whom we share a social identity. In the universe of objects to which humans can emotionally attach, therefore, only other living humans can serve simultaneously as all three types of emotional attachment—a relationship triple-threat. At the same time, emotional attachments to living persons are necessarily the least secure, most fragile, and the most difficult relationships to repair or replace once damaged or lost.

##### 4.3.2.2 External non-persons: tangible and intangible, real and imagined

Our earliest external, non-person attachments are to objects we perceive with our senses and which capture our attention by evoking pleasurable emotions. The richness of warm milk and the coziness of a soft blanket are probably two of most people’s first loves. Initially, of course, infants can’t tell the difference between persons and non-persons, so learning this distinction is a critical early task. As we experience more, and become more discerning in our tastes, we increasingly surround ourselves with physical objects that we love and try to ignore or avoid those we don’t. The feeling of being *at home*, or in harmony with one’s surroundings, results from experiencing the familiar sights, sounds, textures, and smells of cherished objects in one’s environment. The importance of attachments to external things is one reason the destruction of homes and communities in wars or disasters can be so traumatic. We all also form important, enduring attachments to intangible external objects such as languages, ways of life, arts, professions, sports, and other activities. Also in this category are external, non-person objects to which attachment can be destructive, such as abusable substances or the all-too-common love of money and power above all else.

##### 4.3.2.3 Internal persons: internalized images of important others

Through the processes of identification, imitation, and introjection, the infant gradually constructs an internal world in which images of valued, valuing, and kindred human love objects can live on in both declarative (recallable) and procedural memories, to potentially become lifelong sources of nourishment and strength and to form the building blocks of the developing self. Internalized representations of loving caregivers, and of oneself as loved, form the earliest attachment objects in the internal-person quadrant, in the lower left of **Table 2**. Important relationships with family members, teachers, coaches, friends, and others are internalized, as are images of admired heroes from history, literature, or myth. Secure and nourishing internal attachments are likely essential for developing any degree of emotional independence, maturity, and resilience in the face of adversity.

Supplementing currently available external-person attachments with less nourishing but more reliable internal-person attachments seems to be so necessary for moral survival that we all also find nourishment in internal representations of relationships that never existed—that are imagined and held as sustaining and defining fantasies or, as a last resort, incorporated into psychotic delusions. Two apparently universal fantasies are an idealized image of the self, held as a template to become the best version of oneself (dream self), and an image of an ideal love object (dream lover). Internal images of the dream self and dream lover normally evolve over time, modified to become more realistic by each social success and failure, and normally serve as helpful stars by which to navigate the journey to self-actualize. But if one’s currently available attachments to real persons and things, living or dead, are insufficient to sustain selfhood, fantasized or delusive relationships can become a major source of nourishment.

##### 4.3.2.4 Internal non-persons: cherished values, ideals, strengths, and successes

Of the four quadrants in **Table 2**, this one is most difficult to precisely define. It comprises all internalized representations to which one is attached that are not images of other people, an enormous and diverse group of attachments. If one enjoys sufficiently strong and secure attachments in the other three quadrants and has done the work to develop their Wolf and Angel Brains, this quadrant is where cherished values and ideals are held. This is also where everyone’s internal resume is stored, including every personal strength and weakness, and every success and failure. People may pretend—and even convince themselves—that their real resume includes no weaknesses or failures, but even lizards keep honest track of their records of wins and losses. Humans clearly draw emotional strength and self-confidence from their own personal strengths and accomplishments in comparison to others. But everyone’s resilience and self-confidence must also be decremented by their greatest weaknesses and failures, which evoke shame rather than pride, and which, because of their significance, can’t be easily forgiven and forgotten. This quadrant likely has the greatest impact on self-esteem and risk for suicide.

#### 4.3.3 Each person’s set of attachments is unique and constantly changing

It is no accident that the universe of potential human love objects is incredibly diverse and continually expanding. I believe these properties of our object worlds are the fingerprints of biological evolution. I believe we are driven to differ from each other in as many ways as possible because diversity provides the raw material for natural selection, whether of the fittest genes or the fittest solutions to cultural challenges. The breadth, diversity, and continuously changing nature of defining and sustaining emotional attachments also increases the likelihood that every greater good in every culture will eventually be served by someone.

#### 4.3.4 Moral engines grow or shrink depending on the availability of attachments

Of all features of the MEM, the one that most blows my mind is the realization that, perhaps unique among machines, the human moral engine not only speeds up or slows down depending on how much fuel it is fed, but seems to actually grow or shrink, by orders of magnitude, based on the availability of fuel over time. An analogy might be an internal combustion engine that could somehow grow new cylinders just because fuel was plentiful. There seems to be no theoretical limit to how powerful a human moral engine can become as a person becomes an ever-better version of themself, and as their successes add to self-confidence and recruit more supporters. Nor is there likely any limit to how weak a moral engine deprived of sustaining attachments can become, all the way to dead stopped. This plasticity of moral engines also may be part of the phenotypic fail-safe that prevents people who do not enjoy sufficiently broad moral support—such as because of the evil they create—from becoming too powerful. Tragically, it is likely that this same evolutionary fail-safe drives people whose moral engines have slowed too far to experience suicidal impulses. Thomas Joiner’s Interpersonal Theory of Suicide (65), which has received broad research support in several populations, posits that people desire suicide when they experience thwarted belongingness (insufficient sustaining attachments) and perceived burdensomeness (insufficient contributions to the greater good).

## 5 Moral injuries rupture emotional attachments

The MEM predicts that any loss of an important emotional attachment, for any reason, reduces one’s ability to be the best version of themself and contribute meaningfully to the greater good. And every slowing of the moral engine due to reduced attachment availability may then result in secondary losses of other emotional attachments whose affection for us (or ours for them) depended on our ability to engage in specific forms of love, work, play, or creation that may now have become more difficult or impossible. The only thing remaining is to explain how the betrayal of a moral trust, whether by oneself or someone else, can rupture emotional attachments. This is schematically illustrated in **Figure 4**.

**Figure 4:**
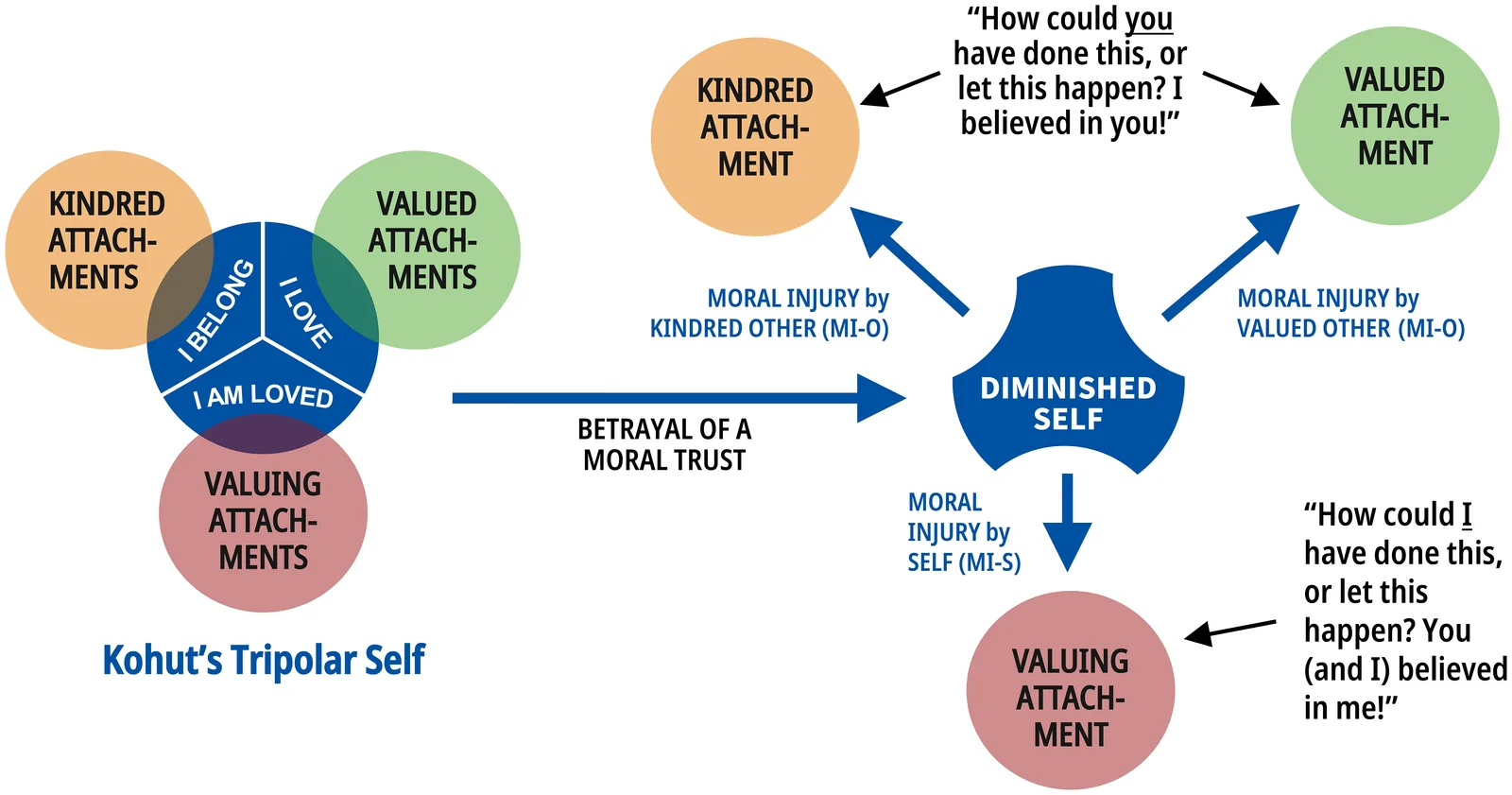
Schematic illustration of the loss of kindred, valued, and valuing attachments as a direct result of the betrayal of a moral trust by oneself or someone else.

On the left side of **Figure 4**, Kohut’s Tripolar Self (64)—core self supported by three types of sustaining attachments—is depicted as four overlapping circles: a central blue circle representing the core self sans attachments (an impossibility for anyone who has lived more than few weeks) overlapped by three circles of different colors representing the self’s universe of currently available valued, valuing, and kindred attachments. The overlap between core self and attachments depicted on the left side of **Figure 4** represents the aspects of the self that are created and maintained only in the space of intimacy and shared experience that defines each unique selfobject relationship. On the right side of **Figure 4**, I have depicted the consequences for the self of a betrayal of a moral trust, whether by a valued or kindred attachment (at the top) or by oneself (at the bottom). In the latter case, valuing attachments may be pushed away and provide less nourishment simply because of shame.

### 5.1 Two moral injury vignettes

#### 5.1.1 “Alice”: an example of moral betrayal by trusted others

As long as she could remember, Alice only ever wanted to be a Marine. Her father and grandfather had both been Marines, and of everyone she knew, these two men were the most capable and self-confident, and seemed to be guided, more than most, by their core moral values. Alice breezed through training, regularly earning the highest scores on academic and physical fitness tests and impressing her instructors with her natural leadership abilities. Alice’s father could not have been prouder watching her graduate from boot camp; he only wished his father was still alive to see Alice become a Marine.

When Alice got to her first duty station, she found being a woman Marine was much harder than she realized. During training, Alice was able to form strong bonds with other women going through the same grueling process, and was mentored by a number of women instructors. At her new duty station, Alice’s section of 16 Marines included only one other woman and she was on her way out, with little interest in befriending Alice. Her leaders were all men. The other Marines rarely made sexual comments around her, but neither did they ever let Alice forget she was not one of them. So Alice was delighted when her section leader, four years and two ranks her senior, invited her to play video games with him in his barracks room that evening. Since Alice wasn’t yet 21, she couldn’t legally drink alcohol in the barracks, but she drank the beer he offered her, nonetheless. She passed out on her section leader’s bed while waiting for her turn at the video game console.

Alice was wakened by her section leader fondling her and attempting to remove her pants, and shrieked as soon as her eyes came into focus. She kicked him away and leaped to her feet, ready to fight, but Alice’s section leader wanted only to calm her down, apologizing and asking her to forget what he had done. Alice left without another word but couldn’t forget. Every time one of the Marines in her section avoided making eye contact with her, and every time she saw them whispering out of her hearing, she was sure they were talking about her. Alice considered reporting the sexual assault, but had heard from too many women Marines that the consequences of crying “rape” in the military are sometimes worse for the victim than the perpetrator, seldom resulting in prosecution.

Over the next few months, Alice’s attitude worsened as she became increasingly angry and depressed. Her hostility toward her section leader leaked out despite her attempts to behave respectfully. One morning, as Alice’s section leader was addressing unit Marines in formation, he loudly berated her for her sloppy appearance, unkempt hair, and wrinkled uniform. When the section leader snarled, “You’ll never be a Marine,” Alice punched him in the eye, breaking a bone in her hand. After the battalion investigated what happened, Alice accepted administrative discharge for “unsuitability” in lieu of prosecution for assault and underage drinking, which would result if she pressed charges against her section leader for sexual assault. Two months later, Alice was a civilian again, but she never told her father or anyone else what really happened. Instead, she concocted a story to explain why she was kicked out of the Marine Corps, a story less shameful and infuriating—and less likely to be traumatic to her father and others—than the truth.

#### 5.1.2 “Bob”: an example of moral betrayal by oneself

Bob was sickly as a young child, making frequent trips to the doctor to manage his asthma and recurrent sinus infections. By the time he was 10, his health had improved, but his experiences of being cared for by this powerful yet gentle healer had already motivated Bob to become a pediatrician himself. To pay for medical school, Bob applied for and received a scholarship from the Navy, which obligated him to serve four years as a Navy medical officer after he graduated. Bob’s younger brother, who was also considering a career in medicine, bragged to everyone about Bob, who was going to be both a doctor and a Naval officer. During medical school and his one-year pediatric internship in the Navy, Bob felt most comfortable managing relatively routine health problems, and least comfortable in situations of urgency and risk of death. So Bob’s wife was alarmed when Bob chose a one-year assignment as a surgeon with a Marine infantry battalion scheduled to deploy to Afghanistan as his obligatory operational tour before continuing his training in pediatrics. Other options offered by the Navy all involved either a longer delay before continuing residency training or having to move his family. Bob assured his wife it would be okay.

After three days of training in advanced trauma life support, Bob was sent to a small outpost in Afghanistan where his battalion was quickly embroiled in combat. Bob was anxious and didn’t sleep well, but held his own until one early morning when a young Marine was brought into the aid station with a penetrating chest wound caused by rocket fragments. Bob correctly diagnosed a collapsed lung, and thought he had correctly inserted a chest tube in an attempt to reinflate it—between the ribs, inside the chest cavity, between the collapsed lung and the chest wall—but in fact, he had inserted the chest just outside the chest cavity, doing nothing. Bob got an x-ray to check the placement of the chest tube, but in his haste and fatigue, misread it, and thinking his patient was stabilized, released him for medical evacuation to Germany, where he would receive definitive care.

Bob learned that afternoon that his patient had died on the flight to Germany of a tension pneumothorax, a death that would have been prevented had Bob correctly placed the chest tube in the battalion aid station. Bob was shocked, then broke down crying after he heard the news. He wanted to contact the deceased Marine’s family to apologize to them, but Bob’s supervisor talked him out of it. Bob soldiered on but increasingly became overwhelmed with anxiety when combat casualties came in, and for the first time since he was ten, experienced full-blown asthma attacks. Between panic attacks and wheezing, Bob could no longer do his job in Afghanistan and so was sent home for treatment and disposition. Realizing his chances to receive further residency training in the Navy were now slim, Bob agreed to a medical discharge without compensation, given that his asthma began in childhood. Bob was devastated by this massive failure, but never told his wife, younger brother, or anyone else in his family about the Marine who died because of his mistake. He was too ashamed. Bob’s wife did her best to remain supportive as their family navigated the stress of transitioning out of the Navy, especially given her husband’s limited future career options, but Bob was sure she never loved him the same after that.

### 5.2 Moral mapping: visualizing emotional attachments

The above two stories of moral injury hint at which attachments may have been damaged or lost because of betrayals of trust, but to fully assess the damage to the self in each case requires a methodical, focused inquiry. One tool that can aid in this exploration is *moral mapping*: a modification of a graphical approach used to visualize social relationships (66). **Figures 5** and **6** depict which of Alice and Bob’s emotional attachments may have been damaged or destroyed, directly or indirectly, because of their moral injuries. The predominant negative moral emotion associated with each now-damaged attachment is represented by the color of its oval and connecting line—in this case, red for anger, blue for shame, and plum for a mixture of anger and shame.

**Figure 5:**
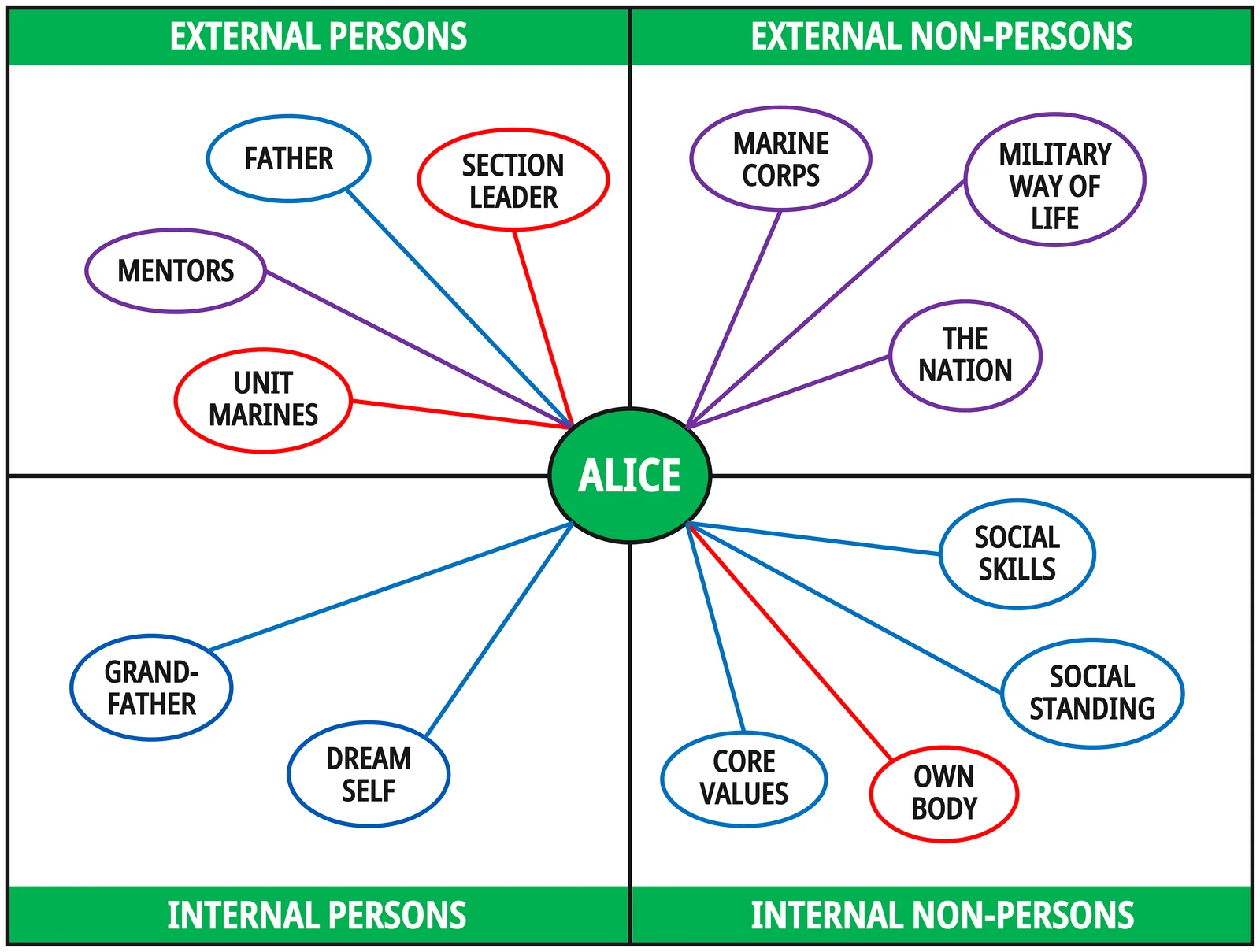
Moral map of defining and sustaining emotional attachments that might have been damaged by Alice’s sexual assault trauma and discharge from the Marine Corps. (Attachments in red are those now affected by Alice’s anger, those in blue by her shame, and those in plum by a mixture of anger and shame).

**Figure 6:**
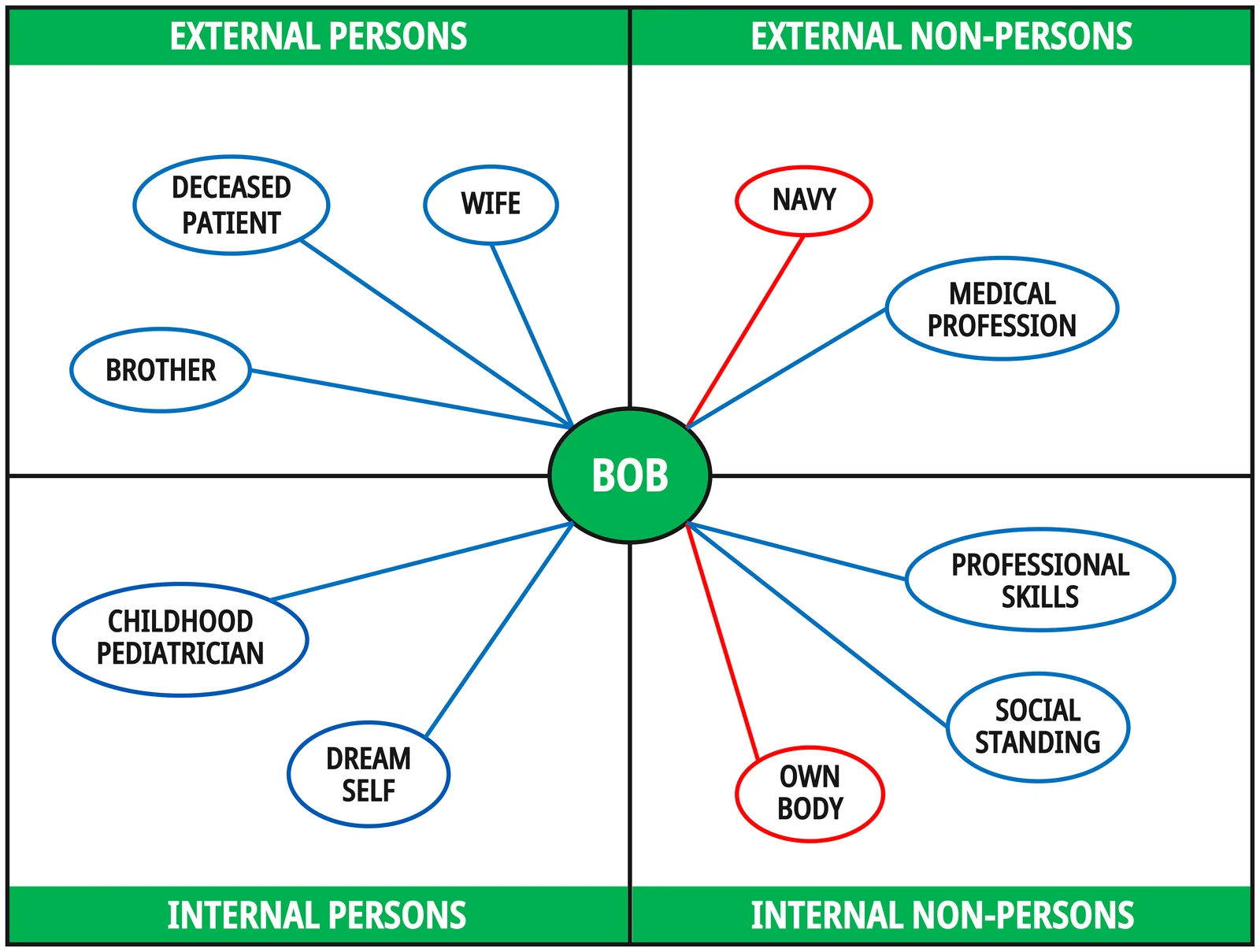
Moral map of defining and sustaining emotional attachments that might have been damaged by Bob’s medical error and discharge from the Navy. (Attachments in red are those now affected by Bob’s anger, and those in blue by his shame).

#### 5.2.1 Alice’s possibly damaged or lost emotional attachments

What were the betrayals of trust, and the consequences of those betrayals, in Alice’s story? First, the Marine Corps failed Alice by sending her to a unit that contained very few women, thus ensuring she would suffer isolation and loneliness and have difficulties adapting. Given her lifelong love for the Marine Corps, Alice’s attachment to it would likely become tinged with both anger (for their failing her) and shame (for her not succeeding as a Marine when other women do), but not severed. She may experience similar mixtures of anger and shame toward the nation and the military way of life.

Next was the betrayal of Alice by the other Marines in her section, likely resulting in unmitigated anger toward them. They were morally obligated to accept Alice and make her feel safe, and they and Alice both knew it. Of course, Alice’s most massive moral betrayal was that perpetrated by her section leader, for not only assaulting her, but then failing to own up to and make amends for his transgression. Her anger for him would likely be unmitigated, and could progress into hatred, causing Alice’s section leader to live on, potentially forever, in her internal world as not a sustaining attachment but a depleting one—an internal relationship that will bring pain and loss of function every time it is activated, the opposite of the pleasure, well-being, and enhanced function characteristic of an activated sustaining attachment.

Alice then experienced a number of impactful secondary betrayals of trust, about which she might be less consciously aware. First among these is Alice’s betrayal of the trust placed in her by her father and grandfather, resulting in her possibly devaluing any support offered by her father because of her shame for having failed them. Alice’s investment in her dream self—the ideal person she dreams of becoming—would also be breached because of her failure as a Marine. Perhaps most deleterious for her health and wellbeing are the betrayals of trust depicted in the lower right of **Figure 5**, including Alice’s likely shame and loss of trust in her own social skills and ability to work in a male-dominated world, her shame over the loss of social standing caused by her sexual assault and failure as a Marine (even if no one else knew about it), and her failure to live up to the core values she learned from her father. She might even abandon her values as impractical and naïve. Her relationship with her own body might also be damaged, causing her to feel anger toward her body for attracting a sexual assault, and for failing to effectively manage the stress that followed.

#### 5.2.2 Bob’s possibly damaged or lost emotional attachments

All effective healthcare providers attach emotionally to their patients, even if only briefly; that’s what it means to care. So Bob would likely experience intense shame over his failure to save the life of his wounded patient. As a result of this failing, Bob might also lose confidence in his professional skills and faith in his dream self to the extent his medical error, health problems, and subsequent discharge from the Navy might prevent him from achieving his life goal of becoming a pediatrician. Bob might feel like he betrayed the trust of his wife and younger brother, and had failed to live up to the internal standard of excellence set by his childhood pediatrician. He would also feel his social standing had been reduced because of his failures as a physician and naval officer, regardless of who knew about these failures.

Even though Bob’s primary morally injurious event involved his betrayal of his own moral expectations, he might eventually feel anger toward the Navy for sending him to a war zone to resuscitate seriously injured men and women without giving him sufficient training to reliably succeed, and then for discharging him without compensation after sustaining a psychiatric injury while serving. He also might feel angry with his own body for breaking under the stress, resulting in the recurrence of his asthma and new-onset panic attacks.

### 5.3 Consequences of attachments being damaged or lost due to moral injury traumas

However hard they would try to forget, both Alice and Bob might reexperience their traumatic events in nightmares and intrusive recollections, reliving the vivid anger and shame they felt at the moment of their betrayals of moral trust, and possibly reexperiencing dissociation as well. They might also experience additional anger and shame in response to whatever secondary failings resulted from their moral injury-induced impairments. Both might also soon encounter the other primary symptom of a moral injury trauma: a loss of trust and diminished ability to form attachments of the same type as were damaged by the moral injury event. For Bob, that might be reflected in impairment in his ability to appropriately attach to his patients and to earn their trust, or to believe in himself as a healer. Alice, on the other hand, might lose trust in her ability to thrive in a male-dominated world, and possibly even to participate in mutually satisfying sexual relationships. Under the weight of potentially worsening depression, anxiety, and social and occupational difficulties, both Alice and Bob might experience chronic allostatic load, progressively eroding their health, wellbeing, and abilities to meet current life challenges. Their personalities might change, becoming more reserved, less affectionate or joyful, and more prone to anxiety, anger, and loss of self-control. Judith Herman coined the term *complex post-traumatic stress disorder* to refer to this constellation of biological, emotional, social, and spiritual symptoms following a severe or prolonged interpersonal trauma ( (67), p. 174).

### 5.4 Tentative principles for clinical treatment based on the MEM

Since the Moral Engine Model of moral injury has not been empirically validated, no evidence-based approaches for clinical care based on the MEM yet exist. In this section I offer a tentative set of brief thoughts about clinical treatment of moral injuries using the MEM, based on clinical practice guidelines for traumatic stress and my own experience. For the prevention and pre-clinical management of moral injuries in occupational or community settings, I recommend the U.S. Marine Corps’ current Combat and Operational Stress Control (COSC) system (68), which includes a toolkit for Stress First Aid that has been widely adapted and repeatedly evaluated (e.g., (69)).

#### 5.4.1 Goals of moral repair

The MEM suggests that the ultimate goal of recovering from a moral injury trauma—perhaps much like that of grieving a loss, overcoming a setback, and navigating a transition—is to restore, enhance, and protect the functioning of a stressed moral engine. Until moral engine function returns, a secondary goal may be to limit collateral moral harm caused by failures to meet ongoing social and occupational responsibilities that may now be impossible. The MEM predicts that successful moral repair will correlate with the extent to which ruptured sustaining attachments are repaired or replaced, new depleting attachments are weakened, and cohesion of the self is increased. Fortunately, I believe, our DNA has known about our vulnerability to moral injury much longer than we have, such that we have evolved innate mechanisms for moral self-repair, given adequate resources, just as our bodies have evolved mechanisms for repair of physical injuries. Discovering which specific practical or emotional resources are needed for moral repair, and gaining access to them, may be important objectives for anyone healing from a moral injury.

#### 5.4.2 Strategies for moral injury treatment

##### 5.4.2.1 Offer to become a temporary valuing sustaining attachment

A sputtering moral engine calls for one thing above all else to get going again: the emotional fuel provided by sustaining attachments. Fortunately, as part of our extended phenotype, we have evolved the capacity to recognize when a fellow human is in distress and to respond instinctively with supportive emotions and behaviors. The importance of a supportive and trustworthy therapeutic relationship for the success of any mental health treatment has been repeatedly documented by research [e.g., (70)]. More to the point of moral repair, hundreds of studies of risk factors for PTSD in adults have documented the crucial role social support, during and after the event, plays as a protective factor promoting recovery from an emotional trauma (47, 71, 72). I argue that of the three types of sustaining attachment defined by the MEM, the one most likely to be helpful to a traumatized patient is a valuing attachment, in which the therapist communicates clearly and often that the patient is cherished and their welfare matters. Serving as a valuing attachment for patient after patient can be hard work, especially when the therapist’s own moral engine is not operating at its best, and losing the ability to genuinely care for patients’ welfare is a prominent symptom of burnout. But few acts may do more to help fellow humans in distress than valuing.

##### 5.4.2.2 Help the patient understand their trauma and its meaning and consequences

Having established a valuing attachment to the patient, the next strategy to consider is to help the patient figure out what, exactly, happened to them, and what resources they need to recover. I believe understanding the trauma requires an appreciation not so much of the events, in their specifics, but of the meaning and consequences of those events. The meaning of an event may refer to one’s understanding of why the event happened and who was responsible for it, but also its significance for one’s understanding of the world and how it works. Adverse consequences, I believe, are what turns experiences of moral distress into literal moral injuries, so assessing the full consequences of a betrayal of moral trust may be an important step toward understanding its social and spiritual significance, and identifying the damage to be repaired. Since both the meaning and consequences of a moral injury tend to evolve and become apparent over time, understanding the trauma can be a protracted and gradual process.

This collaborative exploration can be conducted methodically, step by step, or it can unfold organically as other concerns are addressed. As the therapist and patient gain a shared understanding of the nature and consequences of the trauma, the patient’s job is to develop and implement a strategy for gaining and using the resources needed for moral repair, while the therapist acts as a detective, sounding board, and cheer leader.

##### 5.4.2.3 Identify and remove obstacles to full functioning

The next priority for moral repair may be to identify and remove obstacles to recovery and full functioning. Among the most common and serious obstacles for recovery from a moral injury trauma are those that directly impair the cohesion and wholeness of the self, such as symptoms of dissociation, fragmentation, or emotional dyscontrol, including persistent depression or hypomania, generalized anxiety, panic attacks, rage outbursts, or dissociative flashbacks. Any of those may make the job of restoring old attachments or embracing new ones more difficult. Other obstacles to address include the patient’s current load of social and occupational stressors, which may benefit from being lightened for a time, and the abuse of substances. Potential strategies for removing obstacles include psychotropic medications and behavioral interventions of all types.

##### 5.4.2.4 Repair or replace damaged or lost sustaining attachments

Reaching an understanding of how a morally injurious experience has altered one’s world of external and internal love objects does little to repair the damage. Repairing or replacing damaged or lost internal and external sustaining attachments—and weakening or severing depleting attachments—comprises the major work of moral repair, I believe. Among resources that are potentially helpful for moral repair, one to always consider is the offering or seeking of amends and restitution. For recovery from the betrayal of one’s own trust, such as by a failure to meet a moral obligation, the individual can decide for themselves whether to attempt to right the wrong they created through restitution, and if so, in what form. Often, one cannot undo what was done, so restitution may be more symbolic than literal. The situation is far worse for recovery from moral betrayals perpetrated by others, especially if the perpetrators don’t acknowledge and own up to what they have done, which is too often the case (73).

##### 5.4.2.5 Help accessing other needed resources

Since each instance of moral injury is unique in many ways, the specific resources that are needed to restore moral engine function are also highly individual. Included may be practical resources like financial assistance or help meeting other obligations, educational resources, and social support resources of many types.

## 6 Discussion

A novel integrative theory about the nature of moral injuries has been presented, grounded in evolutionary biology and supported by novel models of innate morality and personhood in the service of cultural evolution. By tracing the evolution of emotions and their associated social capacities in vertebrates, a hierarchy of four concurrent modes of moral reasoning was postulated, serving four simultaneous evolved moral imperatives: survive, acquire social power, love and belong, and seek the greater good. Lifelong requirements for brain and emotional development and vulnerabilities of brain function to deteriorate under acute or chronic stress were recognized as two reasons for variability in biological, emotion-driven morality. Controversies about the nature of peritraumatic dissociation were briefly reviewed and the dissolution theory of Herbert Spencer and John Hughlings Jackson was singled out as the model of dissociation most consistent with current biology.

In order to identify the parts of a person that may be harmed by the betrayal of a moral trust, a broad theory of personhood called the Moral Engine Model was described, based on Maslow’s Hierarchy of Needs theory and an extrapolation to normal people of Heinz Kohut’s findings in narcissistic personalities regarding the role of three types of emotional attachments in maintaining functional integrity of the self, here named valued, valuing, and kindred. Moral injury was then hypothesized to represent rupturing of defining and sustaining, internal or external emotional attachments directly resulting from serious betrayals of moral trust. Two moral injury vignettes were presented, for which damaged internal and external attachments were illustrated using moral mapping techniques. Finally, brief thoughts about the prevention and treatment of moral injury traumas were presented.

Empirically testing the assertions of the MEM may be challenging since few valid instruments yet exist for measuring key aspects of the model, such as the quantity and quality of nourishment received from defining and sustaining internal and external emotional attachments, cohesion of the self, current load of acute and chronic stress, degree of functional connectivity across brain levels, levels of social and spiritual engagement, or social self-esteem. Implementing the MEM as a clinical paradigm may be difficult to the extent the model diverges from popular conceptions of human behavior as always intended, and of the mind as impervious to stress and driven by conscious thought in all situations. Treating moral injuries as literal wounds to the self resulting from failures to uphold innate moral imperatives may require a more humanistic approach to psychological care than is currently popular, such as that described by Carl Rogers, a colleague of Heinz Kohut’s at the University of Chicago in the 1950s and 60s (74). I believe efforts needed to evaluate and implement the MEM, not only in clinical settings but everywhere theories of personhood pertain, will be amply repaid by a fuller understanding of humanness.
